# *N* ^1^,*N* ^2^-Bis[(2-chloro-6-methyl­quinolin-3-yl)methyl­idene]ethane-1,2-diamine

**DOI:** 10.1107/S1600536810041309

**Published:** 2010-10-20

**Authors:** R. Prasath, P. Bhavana, Anand M. Butcher, Ray J. Butcher, Jerry P. Jasinski

**Affiliations:** a Chemistry Group, BITS, Pilani – K. K. Birla Goa Campus, Goa, India 403 726; b Department of Chemistry, Howard University, 525 College Street NW, Washington DC 20059, USA; c Department of Chemistry, Keene State College, 229 Main Street, Keene, NH 03435-2001, USA

## Abstract

The title mol­ecule, C_24_H_20_Cl_2_N_4_, lies on an inversion center in an extended *trans* conformation. In the crystal, weak C—H⋯Cl inter­actions connect the mol­ecules into chains along [010].

## Related literature

For general background to Schiff bases, see: Schiff (1864 ▶); Huiyan *et al.* (2009 ▶); Kano *et al.* (2003 ▶); Liu *et al.* (2010 ▶); Salhi *et al.* (2009 ▶);  Wang *et al.* (2008 ▶); Yong & Zheng (2009 ▶). For related structures, see: Assey *et al.* (2010 ▶); Dipesh *et al.* (2007 ▶).
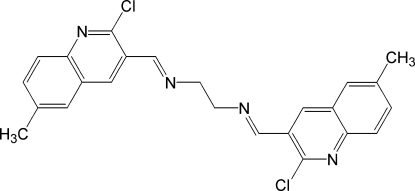


## Experimental

### 

#### Crystal data

C_24_H_20_Cl_2_N_4_
*M* *_r_* = 435.34
Triclinic, 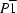

*a* = 4.4088 (8) Å
*b* = 7.2008 (11) Å
*c* = 16.9383 (18) Å
α = 84.236 (11)°
β = 87.924 (12)°
γ = 78.698 (14)°
*V* = 524.57 (14) Å^3^
*Z* = 1
Cu *K*α radiation
μ = 2.93 mm^−1^
*T* = 295 K
0.46 × 0.37 × 0.15 mm

#### Data collection

Oxford Diffraction Xcalibur diffractometer with Ruby Gemini detector
Absorption correction: multi-scan (*CrysAlis PRO*; Oxford Diffraction, 2007 ▶) *T*_min_ = 0.378, *T*_max_ = 1.000
3137 measured reflections
2011 independent reflections
1710 reflections with *I* > 2σ(*I*)
*R*_int_ = 0.028

#### Refinement

*R*[*F* ^2^ > 2σ(*F* ^2^)] = 0.056
*wR*(*F* ^2^) = 0.168
*S* = 1.05
2011 reflections
136 parameters
H-atom parameters constrained
Δρ_max_ = 0.44 e Å^−3^
Δρ_min_ = −0.29 e Å^−3^

### 

Data collection: *CrysAlis PRO* (Oxford Diffraction 2007 ▶); cell refinement: *CrysAlis PRO*; data reduction: *CrysAlis PRO*; program(s) used to solve structure: *SHELXS97* (Sheldrick, 2008 ▶); program(s) used to refine structure: *SHELXL97* (Sheldrick, 2008 ▶); molecular graphics: *SHELXTL* (Sheldrick, 2008 ▶); software used to prepare material for publication: *SHELXTL*.

## Supplementary Material

Crystal structure: contains datablocks I, New_Global_Publ_Block. DOI: 10.1107/S1600536810041309/lh5149sup1.cif

Structure factors: contains datablocks I. DOI: 10.1107/S1600536810041309/lh5149Isup2.hkl

Additional supplementary materials:  crystallographic information; 3D view; checkCIF report

## Figures and Tables

**Table 1:** Hydrogen-bond geometry (Å, °)

*D*—H⋯*A*	*D*—H	H⋯*A*	*D*⋯*A*	*D*—H⋯*A*
C3—H3*A*⋯Cl^i^	0.93	2.86	3.780 (2)	170

Symmetry code: (i) 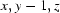.

**Fig. 1.**
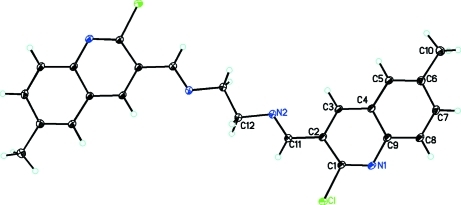
The molecular structure of the title compound with unique part of molecule labeled. Ellipsoids drawn at 30% probability level. The unlabeled atoms are related by the symmetry operator (-x, 1-y, -z).

**Fig. 2.**
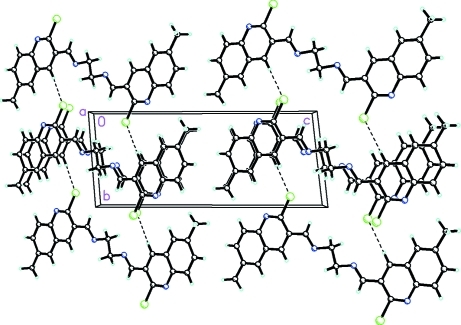
Part of the crystal structure viewed along the a axis showing the intermolecular C—H···Cl interactions as dashed lines.

## supplementary crystallographic information

### Comment

Quinoline Schiff base complexes are important class of compounds owing to their applications in the fields of environmental (Salhi *et al.*, 2009), catalytic (Kano *et al.*, 2003), DNA binding (Yong *et al.*, 2009) and polymeric applications (Huiyan *et al.*, 2009). Quinoline appended Schiff base complexes are also known for their photophysical properties (Liu *et al.*, 2010; Wang *et al.*, 2008). Related structures have already appeared in the literature (Assey *et al.*, 2010; Dipesh *et al.*, 2007).  Herein we report the synthesis and crystal structure of the title compound, (I).

In the title compound, C_24_H_20_Cl_2_N_4_, the molecule is in an extended *trans* conformation and is located on a center of inversion between C12 and C12(-x, 1-y, -z). In the crystal structure, weak C—H···Cl interactions connect molecules into chains along [010].

### Experimental

A mixture of 2-chloro-3-formyl-6-methylquinoline (0.2 g, 1 m*M*) and ethylenediamine (0.03 ml, 0.5 m*M*) was stirred in dichloromethane for 3 h at room temperature. The solvent from the reaction mixture was removed under reduced pressure, and the resulting solid was dried and purified by column chromatography using a 1:3 mixture of ethyl acetate and hexane. Recrystallization was by slow evaporation of a dichloromethane solution of (I) which yielded white coloured needle type crystals. *M*.p. 485–487 K. Yield: 83%.

### Refinement

H atoms were placed in geometrically idealized positions and constrained to ride on their parent atoms with a C—H distances of 0.93, 0.96 and 0.97 Å; *U*_iso_(H) = 1.2*U*_eq_(C)  or *U*_iso_(H) = 1.5*U*_eq_(C_methyl_).

### Crystal data

**Table:**

C_24_H_20_Cl_2_N_4_	*Z* = 1
*M**_r_* = 435.34	*F*(000) = 226
Triclinic, *P*1	*D*_x_ = 1.378 Mg m^−^^3^
Hall symbol: -P 1	Cu *K*α radiation, λ = 1.54184 Å
*a* = 4.4088 (8) Å	Cell parameters from 2032 reflections
*b* = 7.2008 (11) Å	θ = 5.3–73.4°
*c* = 16.9383 (18) Å	µ = 2.93 mm^−^^1^
α = 84.236 (11)°	*T* = 295 K
β = 87.924 (12)°	Plate, colorless
γ = 78.698 (14)°	0.46 × 0.37 × 0.15 mm
*V* = 524.57 (14) Å^3^	

### Data collection

**Table:**

Oxford Diffraction Xcalibur diffractometer with Ruby Gemini detector	2011 independent reflections
Radiation source: Enhance (Cu) X-ray Source	1710 reflections with *I* > 2σ(*I*)
graphite	*R*_int_ = 0.028
Detector resolution: 10.5081 pixels mm^-1^	θ_max_ = 73.6°, θ_min_ = 5.3°
ω scans	*h* = −5→5
Absorption correction: multi-scan (*CrysAlis PRO*; Oxford Diffraction, 2007)	*k* = −8→8
*T*_min_ = 0.378, *T*_max_ = 1.000	*l* = −20→21
3137 measured reflections	

### Refinement

**Table:**

Refinement on *F*^2^	Primary atom site location: structure-invariant direct methods
Least-squares matrix: full	Secondary atom site location: difference Fourier map
*R*[*F*^2^ > 2σ(*F*^2^)] = 0.056	Hydrogen site location: inferred from neighbouring sites
*wR*(*F*^2^) = 0.168	H-atom parameters constrained
*S* = 1.05	*w* = 1/[σ^2^(*F*_o_^2^) + (0.1105*P*)^2^ + 0.063*P*] where *P* = (*F*_o_^2^ + 2*F*_c_^2^)/3
2011 reflections	(Δ/σ)_max_ < 0.001
136 parameters	Δρ_max_ = 0.44 e Å^−^^3^
0 restraints	Δρ_min_ = −0.29 e Å^−^^3^

### Special details

**Table:**

Geometry. All e.s.d.'s (except the e.s.d. in the dihedral angle between two l.s. planes) are estimated using the full covariance matrix. The cell e.s.d.'s are taken into account individually in the estimation of e.s.d.'s in distances, angles and torsion angles; correlations between e.s.d.'s in cell parameters are only used when they are defined by crystal symmetry. An approximate (isotropic) treatment of cell e.s.d.'s is used for estimating e.s.d.'s involving l.s. planes.
Refinement. Refinement of *F*^2^ against ALL reflections. The weighted *R*-factor *wR* and goodness of fit *S* are based on *F*^2^, conventional *R*-factors *R* are based on *F*, with *F* set to zero for negative *F*^2^. The threshold expression of *F*^2^ > σ(*F*^2^) is used only for calculating *R*-factors(gt) *etc*. and is not relevant to the choice of reflections for refinement. *R*-factors based on *F*^2^ are statistically about twice as large as those based on *F*, and *R*- factors based on ALL data will be even larger.

### Fractional atomic coordinates and isotropic or equivalent isotropic displacement parameters (Å^2^)

**Table:**

	*x*	*y*	*z*	*U*_iso_*/*U*_eq_	
Cl	0.37544 (18)	1.10262 (8)	0.15067 (4)	0.0756 (3)	
N1	0.7058 (5)	0.8882 (3)	0.25986 (11)	0.0533 (5)	
N2	0.2488 (4)	0.5727 (3)	0.07293 (11)	0.0532 (5)	
C1	0.5343 (5)	0.8821 (3)	0.20006 (13)	0.0500 (5)	
C2	0.4730 (5)	0.7140 (3)	0.17101 (12)	0.0459 (5)	
C3	0.6152 (5)	0.5444 (3)	0.21010 (12)	0.0463 (5)	
H3A	0.5844	0.4305	0.1934	0.056*	
C4	0.8067 (5)	0.5410 (3)	0.27498 (12)	0.0449 (5)	
C5	0.9619 (5)	0.3710 (3)	0.31671 (13)	0.0507 (5)	
H5A	0.9386	0.2546	0.3009	0.061*	
C6	1.1455 (5)	0.3738 (3)	0.37978 (13)	0.0540 (5)	
C7	1.1733 (6)	0.5531 (4)	0.40297 (14)	0.0597 (6)	
H7A	1.2940	0.5570	0.4464	0.072*	
C8	1.0298 (6)	0.7198 (3)	0.36399 (14)	0.0579 (6)	
H8A	1.0559	0.8348	0.3806	0.069*	
C9	0.8427 (5)	0.7190 (3)	0.29892 (12)	0.0470 (5)	
C10	1.3155 (7)	0.1939 (4)	0.42378 (16)	0.0696 (7)	
H10A	1.2724	0.0859	0.4004	0.104*	
H10B	1.2489	0.1886	0.4784	0.104*	
H10C	1.5339	0.1922	0.4206	0.104*	
C11	0.2710 (5)	0.7195 (3)	0.10311 (13)	0.0498 (5)	
H11A	0.1572	0.8358	0.0823	0.060*	
C12	0.0528 (5)	0.5922 (3)	0.00365 (13)	0.0517 (5)	
H12A	0.1677	0.6255	−0.0439	0.062*	
H12B	−0.1257	0.6933	0.0090	0.062*	

### Atomic displacement parameters (Å^2^)

**Table:**

	*U*^11^	*U*^22^	*U*^33^	*U*^12^	*U*^13^	*U*^23^
Cl	0.1104 (6)	0.0382 (4)	0.0809 (5)	−0.0165 (3)	−0.0379 (4)	−0.0011 (3)
N1	0.0706 (11)	0.0410 (10)	0.0537 (10)	−0.0204 (8)	−0.0112 (9)	−0.0083 (7)
N2	0.0627 (11)	0.0470 (10)	0.0525 (10)	−0.0123 (8)	−0.0189 (8)	−0.0078 (8)
C1	0.0654 (13)	0.0386 (11)	0.0499 (11)	−0.0168 (9)	−0.0107 (9)	−0.0064 (8)
C2	0.0540 (11)	0.0426 (11)	0.0454 (10)	−0.0174 (8)	−0.0046 (8)	−0.0085 (8)
C3	0.0602 (12)	0.0397 (10)	0.0450 (10)	−0.0212 (9)	−0.0062 (9)	−0.0086 (8)
C4	0.0556 (11)	0.0415 (10)	0.0421 (10)	−0.0185 (8)	−0.0041 (8)	−0.0068 (8)
C5	0.0639 (13)	0.0427 (11)	0.0497 (11)	−0.0183 (9)	−0.0072 (9)	−0.0063 (9)
C6	0.0629 (13)	0.0520 (13)	0.0494 (11)	−0.0170 (10)	−0.0066 (9)	−0.0024 (9)
C7	0.0728 (14)	0.0613 (14)	0.0507 (12)	−0.0221 (11)	−0.0205 (10)	−0.0085 (10)
C8	0.0767 (15)	0.0493 (12)	0.0550 (12)	−0.0235 (10)	−0.0149 (11)	−0.0132 (9)
C9	0.0592 (11)	0.0425 (11)	0.0444 (10)	−0.0190 (8)	−0.0036 (8)	−0.0097 (8)
C10	0.0848 (17)	0.0598 (15)	0.0646 (15)	−0.0155 (13)	−0.0223 (13)	0.0021 (12)
C11	0.0599 (12)	0.0417 (11)	0.0499 (11)	−0.0125 (9)	−0.0124 (9)	−0.0047 (8)
C12	0.0600 (12)	0.0471 (12)	0.0498 (11)	−0.0117 (9)	−0.0158 (9)	−0.0053 (9)

### Geometric parameters (Å, °)

**Table:**

Cl—C1	1.747 (2)	C6—C7	1.415 (3)
N1—C1	1.295 (3)	C6—C10	1.504 (3)
N1—C9	1.366 (3)	C7—C8	1.361 (4)
N2—C11	1.242 (3)	C7—H7A	0.9300
N2—C12	1.462 (3)	C8—C9	1.401 (3)
C1—C2	1.428 (3)	C8—H8A	0.9300
C2—C3	1.375 (3)	C10—H10A	0.9600
C2—C11	1.473 (3)	C10—H10B	0.9600
C3—C4	1.405 (3)	C10—H10C	0.9600
C3—H3A	0.9300	C11—H11A	0.9300
C4—C5	1.414 (3)	C12—C12^i^	1.508 (4)
C4—C9	1.422 (3)	C12—H12A	0.9700
C5—C6	1.368 (3)	C12—H12B	0.9700
C5—H5A	0.9300		
			
C1—N1—C9	117.53 (17)	C6—C7—H7A	118.8
C11—N2—C12	117.90 (19)	C7—C8—C9	120.3 (2)
N1—C1—C2	126.0 (2)	C7—C8—H8A	119.9
N1—C1—Cl	115.36 (15)	C9—C8—H8A	119.9
C2—C1—Cl	118.65 (16)	N1—C9—C8	119.18 (18)
C3—C2—C1	116.02 (18)	N1—C9—C4	122.22 (19)
C3—C2—C11	121.40 (18)	C8—C9—C4	118.6 (2)
C1—C2—C11	122.6 (2)	C6—C10—H10A	109.5
C2—C3—C4	120.86 (18)	C6—C10—H10B	109.5
C2—C3—H3A	119.6	H10A—C10—H10B	109.5
C4—C3—H3A	119.6	C6—C10—H10C	109.5
C3—C4—C5	123.30 (18)	H10A—C10—H10C	109.5
C3—C4—C9	117.38 (19)	H10B—C10—H10C	109.5
C5—C4—C9	119.33 (18)	N2—C11—C2	121.6 (2)
C6—C5—C4	121.50 (19)	N2—C11—H11A	119.2
C6—C5—H5A	119.3	C2—C11—H11A	119.2
C4—C5—H5A	119.3	N2—C12—C12^i^	109.9 (2)
C5—C6—C7	117.9 (2)	N2—C12—H12A	109.7
C5—C6—C10	121.9 (2)	C12^i^—C12—H12A	109.7
C7—C6—C10	120.2 (2)	N2—C12—H12B	109.7
C8—C7—C6	122.4 (2)	C12^i^—C12—H12B	109.7
C8—C7—H7A	118.8	H12A—C12—H12B	108.2
			
C9—N1—C1—C2	0.3 (4)	C10—C6—C7—C8	178.5 (2)
C9—N1—C1—Cl	−178.07 (16)	C6—C7—C8—C9	0.9 (4)
N1—C1—C2—C3	−1.1 (4)	C1—N1—C9—C8	−179.9 (2)
Cl—C1—C2—C3	177.21 (16)	C1—N1—C9—C4	1.2 (3)
N1—C1—C2—C11	179.1 (2)	C7—C8—C9—N1	−178.8 (2)
Cl—C1—C2—C11	−2.6 (3)	C7—C8—C9—C4	0.1 (4)
C1—C2—C3—C4	0.4 (3)	C3—C4—C9—N1	−1.8 (3)
C11—C2—C3—C4	−179.78 (19)	C5—C4—C9—N1	178.25 (19)
C2—C3—C4—C5	−179.1 (2)	C3—C4—C9—C8	179.34 (19)
C2—C3—C4—C9	0.9 (3)	C5—C4—C9—C8	−0.6 (3)
C3—C4—C5—C6	−179.8 (2)	C12—N2—C11—C2	−177.51 (19)
C9—C4—C5—C6	0.2 (3)	C3—C2—C11—N2	−8.3 (3)
C4—C5—C6—C7	0.8 (3)	C1—C2—C11—N2	171.5 (2)
C4—C5—C6—C10	−179.0 (2)	C11—N2—C12—C12^i^	−156.5 (2)
C5—C6—C7—C8	−1.3 (4)		

Symmetry codes: (i) −*x*, −*y*+1, −*z*.

### Hydrogen-bond geometry (Å, °)

**Table:**

*D*—H···*A*	*D*—H	H···*A*	*D*···*A*	*D*—H···*A*
C3—H3A···Cl^ii^	0.93	2.86	3.780 (2)	170

Symmetry codes: (ii) *x*, *y*−1, *z*.
